# Improved genetic prediction of the risk of knee osteoarthritis using the risk factor-based polygenic score

**DOI:** 10.1186/s13075-023-03082-y

**Published:** 2023-06-12

**Authors:** Yugo Morita, Yoichiro Kamatani, Hiromu Ito, Shiro Ikegawa, Takahisa Kawaguchi, Shuji Kawaguchi, Meiko Takahashi, Chikashi Terao, Shuji Ito, Kohei Nishitani, Shinichiro Nakamura, Shinichi Kuriyama, Yasuharu Tabara, Fumihiko Matsuda, Shuichi Matsuda, on behalf of the Nagahama study group

**Affiliations:** 1 Department of Orthopedic Surgery, Kyoto University Graduate School of Medicine, Kyoto, Japan; 2 Center for Genomic Medicine, Kyoto University Graduate School of Medicine, Kyoto, Japan; 3 Department of Orthopedic Surgery, Kurashiki Central Hospital, Kurashiki, Japan; 4 Laboratory for Bone and Joint Diseases, Center for Genomic Medicine, RIKEN, Tokyo, Japan; 5 Laboratory for Statistical and Translational Genetics, RIKEN Center for Integrative Medical Sciences, Yokohama, Japan; 6 Department of Orthopedic Surgery, Shimane University Faculty of Medicine, Izumo, Japan; 7 Graduate School of Public Health, Shizuoka Graduate University of Public Health, Aoi-Ku, Shizuoka, Japan

**Keywords:** Polygenic risk score, Knee osteoarthritis, Genetics, Multi-population, Multi-traits

## Abstract

**Background:**

Polygenic risk score (PRS) analysis is used to predict disease risk. Although PRS has been shown to have great potential in improving clinical care, PRS accuracy assessment has been mainly focused on European ancestry. This study aimed to develop an accurate genetic risk score for knee osteoarthritis (OA) using a multi-population PRS and leveraging a multi-trait PRS in the Japanese population.

**Methods:**

We calculated PRS using PRS-CS-auto, derived from genome-wide association study (GWAS) summary statistics for knee OA in the Japanese population (same ancestry) and multi-population. We further identified risk factor traits for which PRS could predict knee OA and subsequently developed an integrated PRS based on multi-trait analysis of GWAS (MTAG), including genetically correlated risk traits. PRS performance was evaluated in participants of the Nagahama cohort study who underwent radiographic evaluation of the knees (*n* = 3,279). PRSs were incorporated into knee OA integrated risk models along with clinical risk factors.

**Results:**

A total of 2,852 genotyped individuals were included in the PRS analysis. The PRS based on Japanese knee OA GWAS was not associated with knee OA (*p* = 0.228). In contrast, PRS based on multi-population knee OA GWAS showed a significant association with knee OA (*p* = 6.7 × 10^−5^, odds ratio (OR) per standard deviation = 1.19), whereas PRS based on MTAG of multi-population knee OA, along with risk factor traits such as body mass index GWAS, displayed an even stronger association with knee OA (*p* = 5.4 × 10^−7^, OR = 1.24). Incorporating this PRS into traditional risk factors improved the predictive ability of knee OA (area under the curve, 74.4% to 74.7%; *p* = 0.029).

**Conclusions:**

This study showed that multi-trait PRS based on MTAG, combined with traditional risk factors, and using large sample size multi-population GWAS, significantly improved predictive accuracy for knee OA in the Japanese population, even when the sample size of GWAS of the same ancestry was small. To the best of our knowledge, this is the first study to show a statistically significant association between the PRS and knee OA in a non-European population.

**Trial registration:**

No. C278.

**Supplementary Information:**

The online version contains supplementary material available at 10.1186/s13075-023-03082-y.

## Background

Knee osteoarthritis (OA) is a common multifactorial disease that causes major public health problems and is a large economic burden on society [1, 2]. The prevalence of knee OA is approximately 35% in individuals over 65 years of age and has been increasing over the past decades [3, 4]. The conventional risk factors for knee OA include sex (women), obesity, and knee injury [5]. Moreover, knee OA has also been shown to be influenced by genetic factors.

The genetic contribution to OA has been demonstrated in twin studies reporting 45–60% heritability estimates for knee OA [6–8]. Moreover, genome-wide association studies (GWAS) have revealed independent susceptibility loci, including rs143384 in the GDF5 gene, for knee OA [9–16]. A previous extensive GWAS meta-analysis for knee OA in 826,690 participants identified 100 independent OA-associated variants and explained up to 11% of the total heritability of knee OA [17, 18]. The discovery of new variants enables a polygenic risk assessment [19], revealing the polygenic architecture of knee OA [20–22]. A polygenic risk score (PRS) is a practical tool that enhances disease risk prediction by aggregating the effects of various common variants [19]. Applications to clinical practice or screening programs in society by stratifying populations into risk groups are currently being explored. There has been intense research on the prediction of knee OA using the PRS [18, 23]. However, PRS accuracy remains moderate (odds ratio [OR] of 1.3 per SD for the risk of knee OA and an OR of 1.1 per SD for the risk of knee replacement), and its assessment is limited to the European population. The reason for lower PRS accuracy for the non-European population compared to that for the European population is because of the dearth of well-conducted studies in globally diverse populations [24].

This study aimed to investigate the predictive accuracy of PRS based on OA GWAS summary statistics in the Japanese population and to improve the predictive ability using multi-population and multi-trait PRSs based on multi-trait analysis of GWAS (MTAG) [25].

## Materials and methods

### Study population

We used phenotypic and genotypic data from the Nagahama Cohort for Comprehensive Human Bioscience, a community-based prospective cohort study. The Nagahama study included 11,645 middle-aged to older adults recruited from 2007 to 2010 and 2016 as the general population living in Nagahama City, a rural city located in central Japan. A total of 8,559 study individuals participated in the first follow-up from 2012 to 2015. Details of participant recruitment have been previously reported [26]. This study was conducted following the principles of the Declaration of Helsinki and was approved by the ethics committee of Kyoto University Graduate School of Medicine and Nagahama Municipal Review Board (no. 278). Written informed consent was obtained from all participants.

### Radiographic evaluation of knee OA

Of the total participants, those aged ≥ 60 years (*n* = 5,018) from 2012 to 2016 were included in the first surveillance and subjected to further radiographic evaluation of the knees; 1,739 participants declined and the remaining 3,279 participants (65.3%) who agreed to participate were included in the present study. The anteroposterior radiography of the knee joints in a fully extended weight-bearing position was evaluated by a registered orthopedic surgeon and a trained examiner using the Kellgren–Lawrence classification (K–L grade) [27]. Knee OA was defined as a K–L grade ≥ 2.

### Genotyping and sequencing

Genomic DNA was extracted from peripheral blood samples using standard laboratory procedures. Our genotype data comprised two data sets: genotyping data sets obtained by single-nucleotide polymorphism (SNP) arrays (9,077 samples, regardless of the knee OA phenotypes) and whole genome sequencing (WGS) data (1,322 samples). Genotyping by the SNP arrays was conducted using a series of four BeadChip microarrays (Illumina, San Diego, CA, USA), namely Asian Screening Array-24v1-0 (ASA; 5,249 samples), HumanOmni2.5-4v1 (2.5–4; 1,261 samples), HumanOmni5Exome-4v1 (5Exome; 725 samples), and HumanCoreExome-12v1 (CoreExome; 1,842 samples). WGS of 1,322 samples was conducted using an Illumina HiSeq X Ten sequencer (Illumina). Genotype calling was performed with 1,826 other Japanese samples based on the GATK best practice [28] using the GRCh37 human reference genome.

### Genotype quality control

Genotype quality control for SNP arrays was performed for each series of BeadChip arrays. Initially, we excluded a total of 120 samples by discordant sex information (*n* = 3), ethnic background other than Japanese (*n* = 21), and low sample call rate < 99% (*n* = 96). Then, SNPs with low SNP call rate (< 0.99), discordance from the Hardy–Weinberg equilibrium (HWE; *p* < 1 × 10^–6^), and low minor allele count (MAC; < 5) in each array were excluded. Finally, we obtained 5,162 samples and 491,997 SNPs for ASA; 1,239 samples and 1,219,577 SNPs for 2.5–4; 723 samples and 1,714,166 SNPs for 5Exome; and 1,833 samples and 249,717 SNPs for CoreExome. The total number of samples that satisfied the quality control (QC) metrics was 8,957. The details are presented in Supplementary Fig. S1a. Genotype QC for WGS was performed as described above for the SNP array. Initially, we obtained 41,888,202 autosomal biallelic SNPs that were marked as PASS by GATK (bcftools -f PASS -v SNP -M 2). Then, we performed sample QC by removing the high discordance rate to the SNP array (> 0.9), ethnic background other than Japanese, high heterozygosity (> 0.05), singleton (> 0.001), and missing rates for at least one chromosome (> 0.1). Only one sample was excluded from the Nagahama study because of a high missing rate, and 12 were removed from the other Japanese samples for miscellaneous reasons. We then excluded 1,974,460 SNPs with a low call rate (< 0.95), 55,712 SNPs that deviated from the HWE (*p* < 1 × 10^–6^), and 18,818,084 SNPs with low MAC (< 2). Finally, we obtained 3,135 samples, including 1,321 samples from the Nagahama cohort, of 21,039,946 SNPs (Supplementary Fig. S1b).

### Genotype imputation for the SNP array data

Imputation of the quality-controlled SNP genotypes of the array dataset was performed using SHAPEIT2 and Minimac4 [29] in each SNP array using 3,135 quality-controlled WGS samples as a reference panel. SNPs with low imputation quality metrics (*r*^2^ < 0.8) and low minor allele frequency (MAF; < 0.01) in at least one array were excluded from the imputation datasets. The 1,321 overlapping samples were excluded from the imputed genotype dataset, and the genotype of WGS was used instead. Finally, we constructed a genotype dataset of 4,693,074 SNPs from 8,957 samples for further analysis.

### Sample extraction for PRS calculation

Of the 3,269 individuals with radiographic data of the knees, we excluded subjects with a history of rheumatoid arthritis (*n* = 76), outliers in principal component analysis who were defined as more than three interquartile ranges in the first and second principal components (*n* = 89), and encrypted first-degree relatedness (Pi-Hat > 0.25, *n* = 252) [30] (Supplementary Fig. S1c).

### Genotype QC for extracted samples

PRS was calculated for the merged imputation and WGS datasets after QC as follows: (1) SNPs with imputation quality metrics *r*^2^ < 0.8 (12,250,404 SNPs for ASA; 11,074,943 SNPs for 2.5–4; 11,324,741 SNPs for 5Exome; and 13,920,025 SNPs for CoreExome). Thereafter those with MAF < 0.01 (2,947,830 SNPs for ASA; 3,554,382 SNPs for 2.5–4; 3,324,770 SNPs for 5Exome; and 1,784,244 SNPs for CoreExome) were excluded from imputation datasets; and (2) SNPs with MAF < 0.01 (13,985,443 SNPs) were excluded from WGS dataset. We obtained 1,644 samples and 5,841,712 SNPs for ASA; 186 samples and 6,410,621 SNPs for 2.5–4; 119 samples and 6,390,435 SNPs for 5Exome; 274 samples and 5,335,677 SNPs for CoreExome; and 629 samples and 7,054,503 SNPs for WGS dataset. Finally, 4,693,074 variants common to all imputation and WGS datasets were extracted from each dataset to avoid bias due to the different number of variants in the datasets, and all datasets were merged (Supplementary Fig. S1d). Data management and analyses were performed using PLINK 2.0, and data merging was performed using PLINK 1.07 [31].

### PRS calculation based on single traits

We calculated PRS as the sum of alleles associated with a trait weighted by the effect size determined by a previous GWAS using PRS-CS [32] with East Asian linkage disequilibrium (LD) score reference panels from the 1000 Genome Project. We used the ‘auto’ mode of PRS-CS to derive model parameters from a fully Bayesian approach without a validation dataset. The PRS of each individual was calculated based on best-guess genotypes, and only the SNPs found at HapMap3 sites were included to contribute to the score to reduce the computational cost [32, 33].

**Fig. 1:**
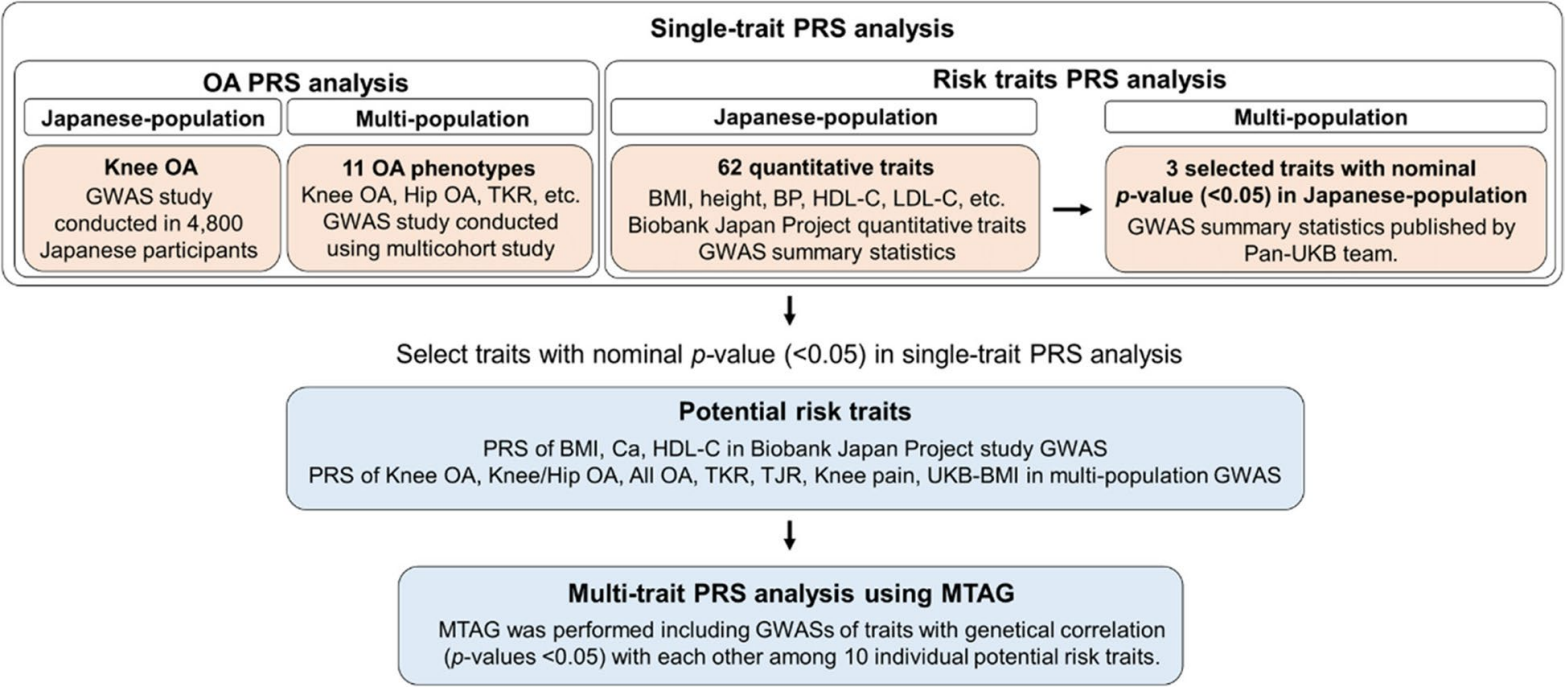
Polygenic risk score analysis flow chart. PRS, polygenic risk score; GWAS, genome-wide association study; OA, osteoarthritis; TKR, total knee replacement; BMI, body mass index; BP, blood pressure; HDL-C, high-density lipoprotein cholesterol; LDL-C, low-density lipoprotein cholesterol; Ca, calcium; TJR, total knee and/or hip joint replacement; UKB, UK Biobank; MTAG, multi-trait analysis of genome-wide association

A flowchart of the PRS analysis is shown in Fig. 1. In the OA PRS calculation, the Japanese knee OA PRS was computed by obtaining the largest Japanese knee OA GWAS summary statistics that identified two genome-wide significant SNPs in the human leukocyte antigen class II/III locus associated with susceptibility to knee OA using 4,800 Japanese participants [9]. To calculate for the larger sample size OA PRS and summary statistics of extensive GWAS meta-analysis for OA (10 different OA phenotypes [17] and knee pain GWAS summary statistics [34]) in 826,690 participants across 13 cohorts worldwide (encompassing Japanese knee OA GWAS [9]) were obtained.

To identify the risk factors for knee OA, GWAS summary statistics of 62 quantitative traits were obtained from the Biobank Japan (BBJ) Project [35–38], and the Japanese single-ancestry PRSs were calculated. We calculated the multi-population PRS [39–41] of the corresponding traits in the MTAG of Japanese single-ancestry GWAS using GWAS summary statistics published in the UK biobank (Pan-UKB team. https://pan.ukbb.broadinstitute.org. 2020.; Supplementary Table S1). Before analysis, SNPs with MAF < 0.01 and imputation quality metrics *r*^2^ < 0.8 were excluded from the GWAS summary statistics.

We also calculated PRSs using LDpred2-auto [42] for Japanese knee OA and traits that were suggested as risk factors for knee OA (PRS-CS analysis, *p* < 0.05). For LDpred2-auto, only the SNPs found at HapMap3 sites were included, and the posterior effect size was computed using 30 initial values for the proportion of causal variants from 1 × 10^–4^ to 0.2 equally spaced on the log scale. The initial heritability value was obtained using constrained LD score regression. After filtering the outlier predictions (> 3 median absolute deviations from their median), we used the mean of the remaining predictions as the final PRS.

### PRS calculation based on multi-traits

We selected GWAS for traits suggested as risk factors for knee OA (PRS analysis, *p* < 0.05). We identified traits that were genetically correlated with each other in the selected traits using the linkage disequilibrium score regression [43]. Traits with a genetic correlation *p*-value < 0.05 were identified as suitable for inclusion in the MTAG. Of these, the traits that were only genetically correlated with each other we incorporated into traits associated with knee OA in the PRS analysis using MTAG (version 1.0.7). East Asian LD score reference panels from the 1000 Genome Project were used for all the analyses.

### Statistical analysis

Statistical analyses were performed using the R statistical software version 4.0.4 (R Foundation for Statistical Computing, Vienna, Austria). The association between PRSs and knee OA in the Nagahama study participants was evaluated using a logistic regression model. We calculated adjusted OR per SD and 95% confidence interval (CI) for the risk of knee OA. Age, sex, 10 principal components, and the type of dataset to which the sample belongs (SNP array or WGS dataset) were included as covariates. Disease liability explained by PRS was estimated by the conversion of observed PVE to the R^2^ on the liability scale using a linear model with disease prevalence in the Nagahama study participants (37.1%) [44]. To evaluate the ability of PRS in case/control discrimination, receiver operating characteristic (ROC) analyses were performed by plotting the true- against the false-positive rate. The area under the curve (AUC) was calculated using a non-parametric method [45]. In addition, to assess the clinical usefulness of the PRS in the prediction of knee OA, the PRS was combined with a currently known clinical risk score and differences in model performance were evaluated. The first model (MODEL I) was constructed using only the clinical risk score of individuals, including sex, age, and body mass index (BMI) [46]. The second model (MODEL II) was generated by incorporating the PRS into MODEL I (sex, age, and BMI). To test the improvement in model performance by incorporating PRS, the AUC test (Delong method) [47] and net reclassification improvement (NRI) analysis [48] were performed. We compared the AUC (c statistic) generated from the full model with the values generated from the model, with each factor removed, to evaluate the relative contribution of each factor to the model. In a sensitivity analysis, we explored whether additional adjustment for excluding statin users would change our findings on lipid traits PRS, high- and low-density lipoprotein cholesterol (HDL-C and LDL-C), total cholesterol, and triglyceride. To assess the ability of PRS in case/control discrimination and its correlation with disease severity, the Jonckheere–Terpstra trend test was used to compare the distributions of radiographic knee OA severity (K-L grade) between different quintiles of PRS. All polygenic scores were standardized to facilitate interpretability. For knee OA risk factor screening to identify traits to include in MTAG, we applied a nominal threshold of *p* = 0.05. We applied the Bonferroni correction for multiple comparisons (number of PRS tested *N* = 19), considering as significant a *p*-value < 0.0026 (0.05/19) in risk factors PRS analysis based on single and multi traits. In a sensitivity analysis excluding statin users, a *p*-value < 0.0125 (0.05/4) was considered significant.

## Results

Of the 2,852 individuals (1,776 women and 1,076 men) included in our final genetic analysis. Among these 2,852 individuals, 1,059 (37.1%) had knee OA and the mean age was 68.2 years ([SD] = 5.3; range, 60–80 years; Table 1). Although the Nagahama cohort study was conducted on volunteers from a rural area in Japan, the prevalence of knee OA was almost perfectly consistent with that of previous studies in other countries [49, 50].

**Table 1:** Demographic data of participants

Sex (female / male)	1776 / 1076
Mean age (years)	68.2 (SD 5.3, 60 to 80)
Mean body mass index (kg/m^2^)	22.5 (SD 3.1, 12.8 to 40)
Kellgren-Lawrence grade	0:110 / 1:1683 / 2:805 / 3:230 / 4:24

*SD* Standard deviation

### Performance of PRSs derived from OA GWAS

We first evaluated the ancestry-specific PRS derived from a Japanese knee OA GWAS (Supplementary Table S1). The PRS was not associated with risk for knee OA (*p* = 0.228). We then evaluated PRS performance based on multi-cohort OA GWAS because increasing evidence has demonstrated larger sample size multi-ancestry PRS improved performance [32, 39–41]. We selected the largest multi-population OA GWAS [17] and found that PRSs derived from multi-population GWAS of knee OA, knee and/or hip OA, OA, total knee replacement (TKR), total knee and/or hip joint replacement (TJP), and knee pain showed nominal associations (*p* < 0.05) with knee OA (Table 2). The strongest association was observed for multi-population knee OA PRS (*p* = 6.7 × 10^–5^, OR per SD increase 1.19 [95% CI 1.09–1.29]). The adjusted OR and R^2^ on the liability scale ranged between 1.10–1.19 and 0.14–0.52%, respectively.

**Table 2:** PRS analysis summary of traits that showed association with knee OA

Traits	*p*-value	Adjusted OR (95% CI)	R^2^ (%)	AUC	AUC test *p*-value	NRI (*p*-value)
**OA PRS analysis**						
**Multi-population GWAS**						
Knee OA	6.70E-05*	1.19 (1.09–1.29)	0.520	0.540	0.034	0.081 (0.036)
Knee and/or hip OA	9.90E-04*	1.15 (1.06–1.25)	0.421	0.534	0.059	0.054 (0.161)
All OA	0.014	1.11 (1.02–1.21)	0.330	0.530	0.067	0.027 (0.486)
Total knee replacement	0.008	1.12 (1.03–1.22)	0.165	0.523	0.120	0.067 (0.085)
Total knee and/or hip replacement	0.025	1.10 (1.01–1.19)	0.144	0.524	0.197	0.068 (0.079)
Knee pain	0.026	1.10 (1.01–1.19)	0.211	0.527	0.254	0.092 (0.017)
**Risk trait PRS analysis**						
**Biobank Japan GWAS**						
Body mass index	4.57E-06*	1.22 (1.12–1.32)	0.689	0.548	0.272	0.054 (0.162)
Calcium	0.021	0.91 (0.83–0.99)	0.072	0.514	0.264	0.086 (0.027)
High-density-lipoprotein cholesterol	6.94E-04*	0.87 (0.80–0.94)	0.434	0.539	0.118	0.116 (0.003)
**Multi-population GWAS**						
UKB-body mass index	0.002*	1.14 (1.05–1.24)	0.523	0.540	0.361	-0.017 (0.658)

*PRS* Polygenic risk score, *OA* Osteoarthritis, *OR* Odds ratio, *AUC* Area under the curve, *NRI* Net reclassification improvement, *GWAS* Genome-wide association study, *UKB* UK Biobank

*P*-values were evaluated using a logistic regression model. Adjusted OR per standard deviation and 95% confidence intervals of risk of knee OA were calculated using a logistic regression model including age, sex, 10 principal components, and a dummy variable for the array type used in genotyping as covariates. Disease liability explained by the PRS was estimated by the conversion of observed PVE to R2 on the liability scale using a linear model. The AUC test (Delong method) and net reclassification improvement analysis were performed to compare MODEL I (only clinical information, including sex, age, and BMI) and MODEL II (incorporating PRS into MODEL I)

^*^Significant after Bonferroni correction

### Performance of PRSs derived from GWAS of the risk factors for OA

We evaluated the association between the PRS of quantitative traits using the summary statistics of BBJ and knee OA to identify potential risk factor traits that may share genetic components with knee OA to be included in the MTAG. High PRS scores for BMI and low scores for HDL-C and calcium (Ca) were nominally associated (*p* < 0.05) with an increased risk of knee OA (Table 2). BMI PRS displayed the strongest association with knee OA (*p* = 4.6 × 10^−6^, OR per SD increase 1.22 [95% CI 1.12–1.32]) and outperformed multi-population knee OA PRS, highlighting the known risk of BMI for knee OA as well as the currently explained heritability of BMI GWAS in contrast to that of OA GWAS. HDL-C PRS showed the second strongest correlation with knee OA (*p* = 6.9 × 10^−4^, OR per SD increase 0.87 [95% CI 0.80–0.94]), suggesting a shared genetic background of knee OA with lipid metabolism. In a sensitivity analysis for lipid traits PRS, 916 statin users were excluded, and 1,936 individuals (683 knee OA patients; 35.3%) were analyzed. After Bonferroni correction, only HDL-C PRS showed significant associations (*p* = 0.004, OR per SD increase of 0.86 [95% CI 0.78–0.95]; Supplementary Table S2). Ca PRS was inversely associated with knee OA (*p* = 6.9 × 10^−4^, OR per SD increase 0.91 [95% CI 0.83–0.99]). We obtained UKB-GWAS data for the above-mentioned quantitative traits and calculated the PRSs. We observed that, despite the larger sample size, multi-ancestry PRSs of the risk factors performed worse than those of the BBJ traits and only the UKB BMI PRS showed nominal associations (*p* = 0.002, OR per SD increase of 1.14 ([95% CI: 1.05–1.24]; Supplementary Table S1). A comprehensive analysis of risk factors suggested that BMI, HDL-C, and Ca have a genetic risk effect on knee OA. The correlation with BMI was particularly strong and remained strong even in the UKB-BMI PRS, which excluded the Japanese population. PRSs estimated using LDpred 2-auto are shown in Supplementary Table 3. The results revealed slightly lower performance in the PRSs computed using LDpred 2-auto than those computed using PRScs; nevertheless, both PRSs using PRScs and LDpred 2-auto showed similar trends. The multi-population knee OA PRS and BBJ BMI PRS were significant after Bonferroni correction in both PRS analyses using PRScs and LDpred2-auto.

### Improved prediction of the knee OA risk using MTAG-based PRS

**Fig. 2:**
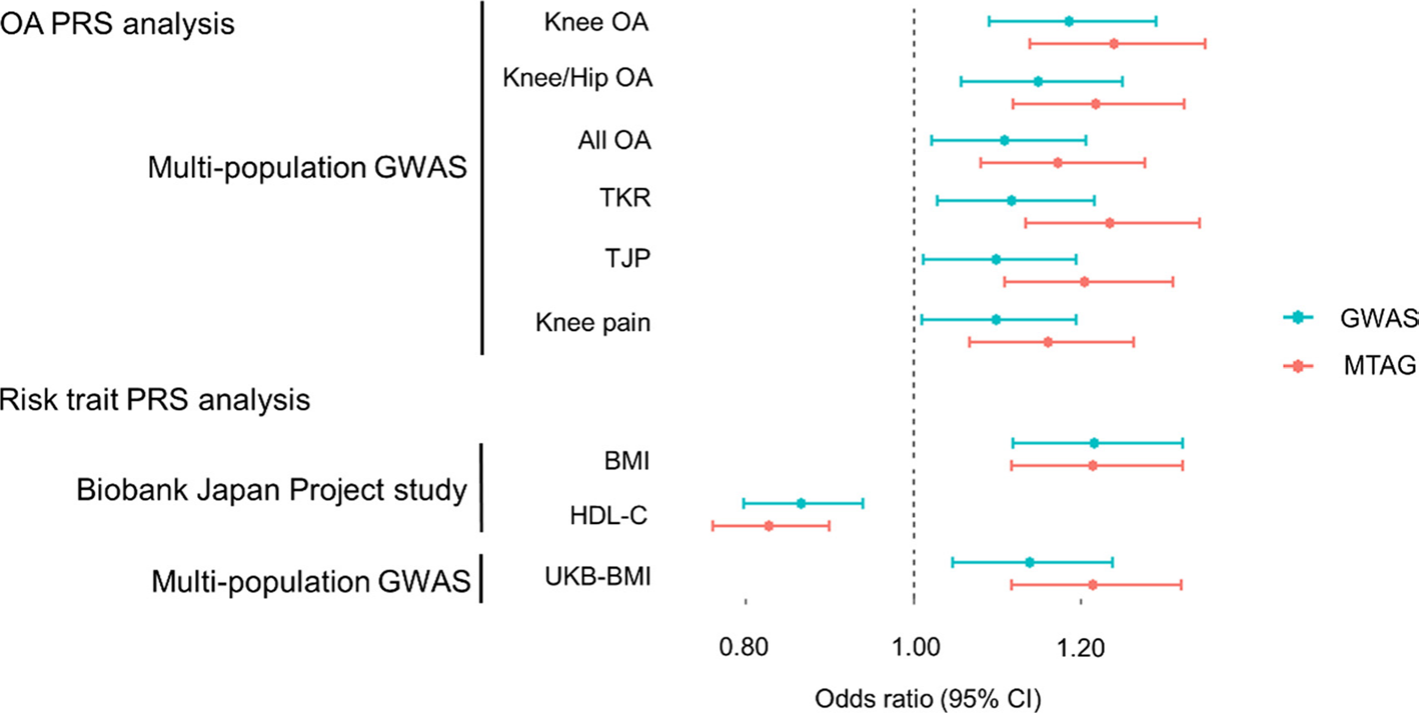
Adjusted odds ratios of single-trait and MTAG-based PRSs of traits with correlations with knee OA. Adjusted odds ratios per standard deviation and 95% confidence intervals of the risk of knee OA were calculated using a logistic regression model, including age, sex, 10 principal components, and a dummy variable for the array type used in genotyping as covariates. PRS, polygenic risk score; MTAG, multi-trait analysis of genome-wide association; OA, osteoarthritis; GWAS, genome-wide association study; TKR, total knee replacement; TJR, total knee and/or hip joint replacement; BMI, body mass index; HDL-C, high-density lipoprotein cholesterol; UKB, UK Biobank; CI, confidence interval

MTAG can increase the statistical power by appropriately incorporating the information in GWAS estimates for other genetically correlated traits [25]. We first selected traits that showed nominal association with knee OA risk in the single-trait PRS analysis to develop MTAG-based PRSs for a better prediction model. We selected 10 traits from the GWAS summary statistics, including six multi-ancestry OA-related traits, three single-ancestry risk factor traits, and a multi-ancestry BMI. Next, we evaluated genetic correlations among the 10 traits (Supplementary Fig. S2) and incorporated traits that were genetically correlated with each other into all 10 traits (Fig. 1). All MTAG-based PRS showed significant associations with knee OA after Bonferroni correction (*p* < 0.0026). Most MTAG-based PRS yielded stronger risk effects with knee OA (Fig. 2) and outperformed R^2^ on the liability scale compared to the corresponding single-trait PRS, with an average of 2.0-fold (Table 3). The PRS with the best performance was the MTAG-based multi-population knee OA PRS, calculated by incorporating BBJ GWAS of BMI, multi-population GWAS of the knee and/or hip OA, all OA, TKR, TJP, and knee pain, and UKB-GWAS of BMI into multi-population knee OA GWAS using MTAG (*p* = 5.4 × 10^−7^, OR per SD increase 1.24 [95% CI 1.14–1.35]).

**Table 3:** MTAG-based PRS analysis summary

Traits	*p*-value	Adjusted OR (95% CI)	R^2^ (%)	AUC	AUC test *p*-value	NRI (*p*-value)
**OA PRS analysis**						
**Multi-population GWAS**						
Knee OA	5.42E-07*	1.24 (1.14–1.35)	0.967	0.559	0.029	0.105 (0.006)
Knee and/or hip OA	3.84E-06*	1.22 (1.12–1.32)	0.837	0.553	0.038	0.089 (0.022)
All OA	1.62E-04*	1.17 (1.08–1.28)	0.598	0.543	0.090	0.060 (0.121)
Total knee replacement	8.61E-07*	1.23 (1.14–1.34)	0.907	0.557	0.041	0.085 (0.028)
Total knee and/or hip replacement	1.22E-05*	1.20 (1.11–1.31)	0.740	0.550	0.049	0.097 (0.012)
Knee pain	4.92E-04*	1.16 (1.07–1.26)	0.407	0.535	0.042	0.062 (0.110)
**Risk trait PRS analysis**						
**Biobank Japan GWAS**						
Body mass index	4.65E-06*	1.21 (1.12–1.32)	0.776	0.554	0.212	0.051 (0.189)
High-density-lipoprotein cholesterol	9.84E-06*	0.83 (0.76–0.90)	0.741	0.551	0.083	0.087 (0.025)
**Multi-population GWAS**						
UKB body mass index	5.07E-06*	1.21 (1.12–1.32)	0.882	0.559	0.177	0.059 (0.130)

*MTAG* Multi-trait analysis of genome-wide association study, *PRS* Polygenic risk score, *OA* Osteoarthritis, *OR* Odds ratio, *AUC* Area under the curve, *NRI* Net reclassification improvement, *GWAS* Genome-wide association study, *UKB* UK Biobank

*P*-values were evaluated using a logistic regression model. Adjusted OR per standard deviation and 95% confidence intervals of risk of knee OA were calculated using a logistic regression model including age, sex, 10 principal components, and a dummy variable for the array type used in genotyping as covariates. Disease liability explained by the PRS was estimated by the conversion of observed PVE to R2 on the liability scale using a linear model. The AUC test (Delong method) and net reclassification improvement analysis were performed to compare MODEL I (only clinical information, including sex, age, and BMI) and MODEL II (incorporating PRS into MODEL I)

^*^Significant after Bonferroni correction

**Fig. 3:**
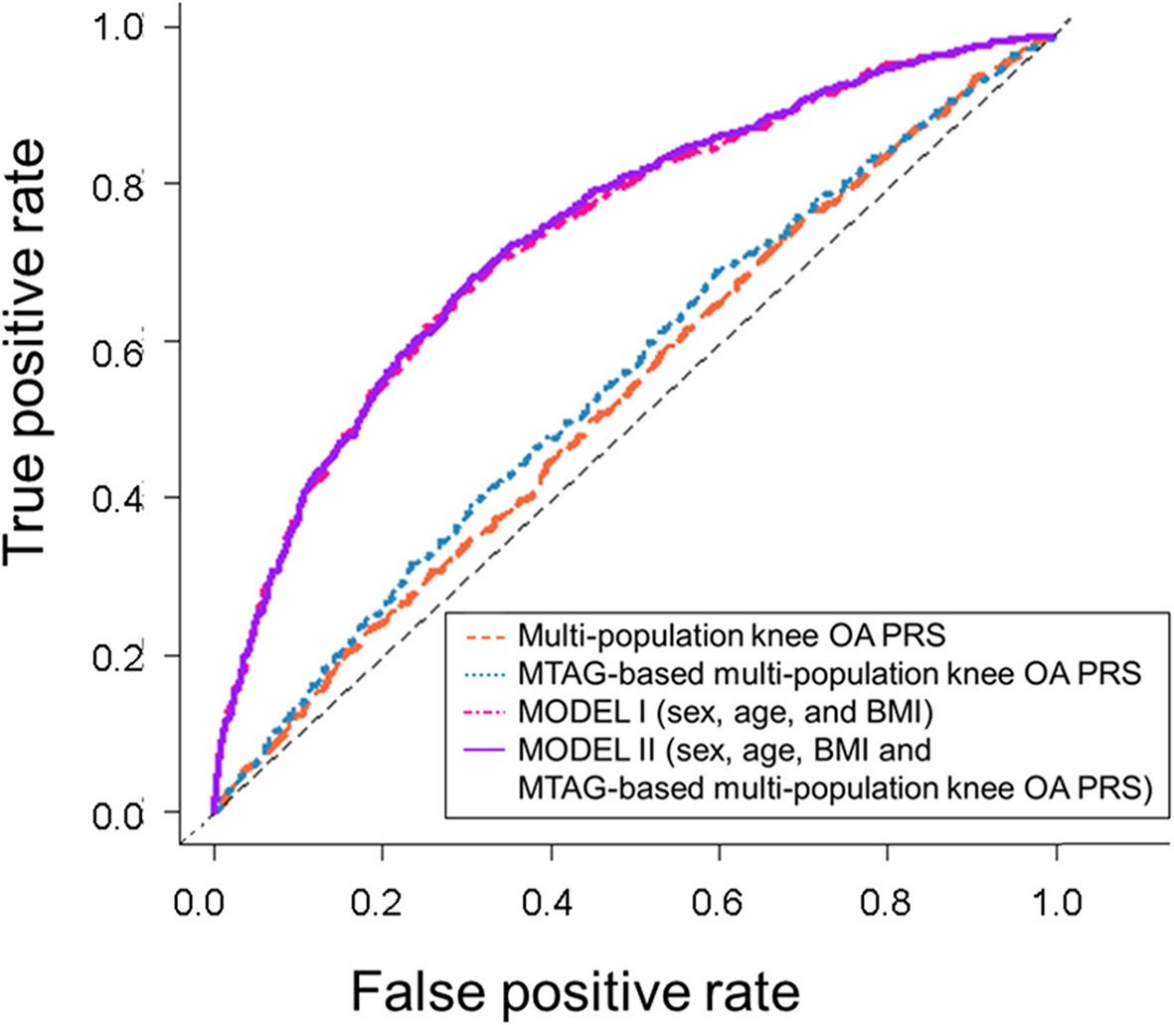
Receiver operating characteristic curves for identifying patients with knee OA using PRSs and clinical data. Line plot showing receiver operating characteristic curves of PRSs and models, including clinical data. MODEL I integrated risk models using clinical data, including sex, age, and BMI; MODEL II integrated risk models using clinical data, including sex, age, and BMI, and MTAG-based multi-population knee OA PRS. OA, osteoarthritis; PRS, polygenic risk score; BMI, body mass index; MTAG, multi-trait analysis of genome-wide association

**Fig. 4:**
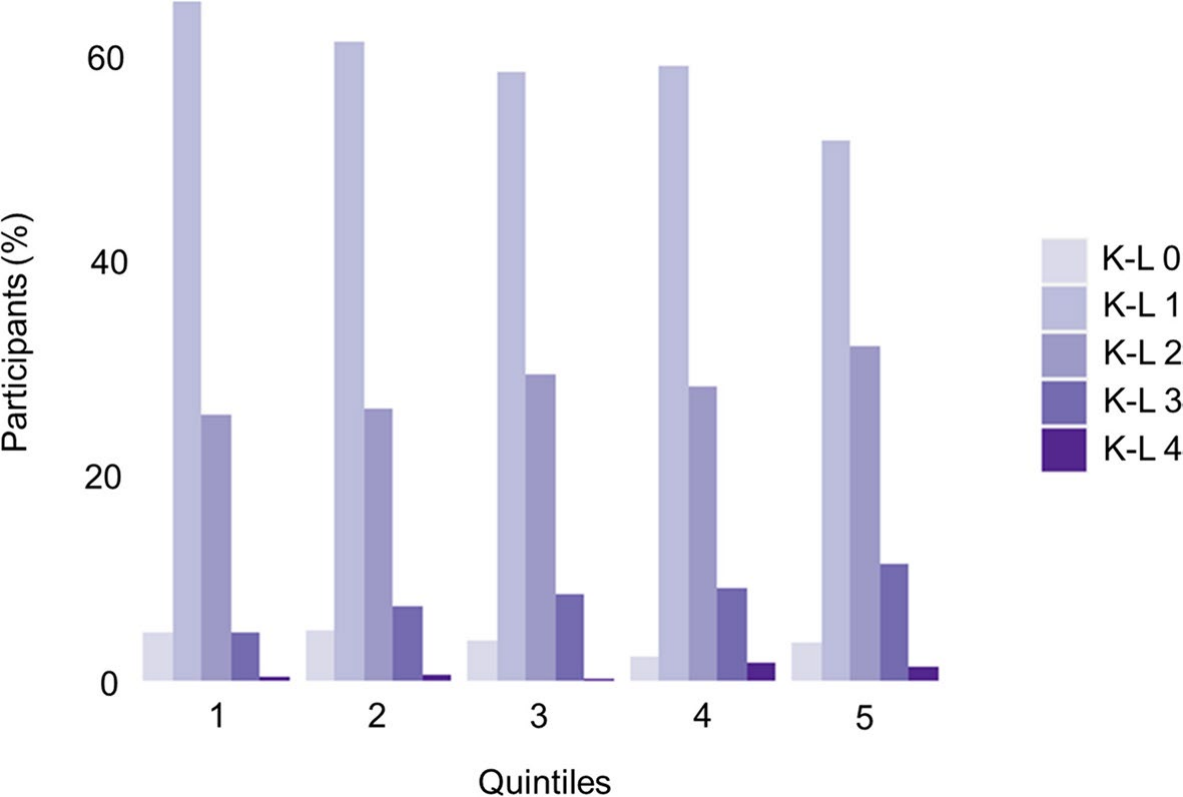
Distribution of K-L grade per quintile of MTAG-based multi-population knee OA PRS. K-L, Kellgren–Lawrence grade; MTAG, multi-trait analysis of genome-wide association; OA, osteoarthritis; PRS, polygenic risk score

To evaluate whether adding the PRS to a clinical risk model improves the overall model performance, we compared MODEL I (constructed with only the clinical risk score of individuals, including sex, age, and BMI) to MODEL II (incorporating PRS into MODEL I; Tables 2 and 3). The model incorporating MTAG-based multi-population knee OA PRS into MODEL I showed the best improvement in AUC (AUC 74.4–74.7%, AUC test *p* = 0.029) and a significant NRI value of 0.105 (95% CI 0.030–0.181, *p* = 0.006). The contributions of each factor were 9.0, 3.6, 3.3, and 0.3% for sex, age, BMI, and MTAG-multi-population knee OA PRS, respectively (Fig. 3). The MTAG-based multi-population knee OA PRS was also correlated with radiographic severity; we observed a strong association between MTAG-based multi-population knee OA PRS and severe OA (K-L 3 and 4; *p* = 1.3 × 10^−5^, OR per SD increase 1.35 [95% CI 1.18–1.55]) and increasing radiographic knee OA severity (high K–L grade) in increasing quintiles of MTAG-based multi-population knee OA PRS (The Jonckheere–Terpstra trend test *p*-value = 3.1 × 10^−7^; Fig. 4).

## Discussion

Knee OA is a multifactorial disorder with a polygenic genetic architecture. Therefore, understanding the genetic etiology of knee OA is challenging. The largest OA GWAS to date performed by the Genetics of OA (GO) consortium, including two East Asian and 11 European cohorts, explained 11% of the genetic risk for knee OA [17]. In the European population, there have been a few previous attempts to predict OA using PRS, and a modest discriminatory ability of knee OA was reported, with an estimated risk effect of the OR of 1.2 for radiographic knee OA [18]. However, the PRS assessment of knee OA has been mostly limited to the European population and the prediction accuracy of PRS in non-Europeans remains limited [24]. In this study, we observed an OR of 1.19 per SD increase in multi-population knee OA PRS in the Japanese population. The slightly higher OR of 1.30 for severe radiographic knee OA shown in the Rotterdam Study [18] was also replicated in the Japanese population (OR per SD increase 1.32 [95% CI 1.15–1.52]). Moreover, the present study demonstrates that the predictive performance of the knee OA PRS can be improved by using multi-population OA GWAS with increased statistical power due to a larger sample size and multi-trait PRS using MTAG, including risk factor traits of knee OA, even when the Japanese knee OA GWAS population is small. The improved performance by multi-population OA GWAS could either be due to a multi-population nature or an increased sample size. In most cases, the MTAG-based PRS outperformed their corresponding single-trait PRSs in predicting knee OA, and our prediction model combining the MTAG-based multi-population knee OA PRS and established clinical risk factors showed better predictive ability than that of clinical risk factors only. PRS calculations were performed using PRScs which assumes the continuous shrinkage priors and LDpred2-auto which assumes a point-normal mixture distribution for SNP effect sizes [32]. Both methods with different concepts in PRS calculations yielded the same results, suggesting the accuracy of the results of this study.

In the present study, the PRS generated based on the GWAS of the Japanese population knee OA (single Japanese ancestry) with a limited sample size was not associated with the prevalence of knee OA, supporting the current concern regarding the difficulty in implementing equitable genomic medicine across non-European populations [24]. In these cases, the use of multi-population and/or multi-trait analysis can improve the predictive ability of PRS. The present study is the first to investigate the increase in the predictive performance of PRS for knee OA in non-European populations. The PRS from a multi-population knee OA GWAS was observed to be moderately accurate in the Japanese population (AUC = 0.540). PRS accuracy and predictive power depend on the power of the base GWAS data, and the multi-population OA GWAS may have increased the predictive accuracy because it represents a meta-analysis study that included the Japanese knee OA GWAS [9, 17] and overwhelmingly increased the sample size, reflecting the multifactorial genetic etiology of knee OA and the additive effect of genes associated with knee OA. Moreover, our results showed that compared to single-trait PRS, multi-trait PRS using MTAG improved the predictive performance by approximately 2.0 times as in R^2^ on the liability scale. The PRS approach is expected to provide insights into genetic etiology, free from confounding bias [24]. Unlike actual measurements, genetic scores do not vary with time and can be evaluated universally; therefore, PRS effectively identifies individuals at a high risk of knee OA. Highly accurate risk prediction allows for focused prevention strategies such as weight reduction, biomechanical interventions such as knee braces, and exercise for high-risk groups [51]. Furthermore, the statistically significant improvements in the predictability of the model by integrating clinical information and PRS suggest its potential clinical applications.

In the present study, PRS analysis using nationwide biobank GWASs of the same population [24] suggested a genetic overlap with knee OA for several traits, including novel traits. The influence of high BMI on the development of knee OA has been reported for both the biomechanical [5, 52] and genetic pathways [14, 53, 54]. In our study, the genetic relationship between high BMI and OA of the knee and weight-bearing joints was consistent and robust. Moreover, a previous epidemiological study has reported that serum HDL-C levels were low in patients with knee OA [55] and that HDL-C levels in the synovial fluid were negatively correlated with cartilage damage and the severity of knee OA [56]. Although the genetic relationship between HDL-C and knee OA is unclear [53, 57], in the present study, HDL-C PRS was strongly negatively correlated with knee OA, second to BMI PRS. A previous Mendelian randomization study for OA has reported a causal relationship between low LDL-C and OA and the potential role of statin in OA pathogenesis [53]. We performed a sensitivity analysis excluding statin users, but only HDL-C PRS was significant after the Bonferroni correction. It is possible that the sample size was limited and the significance of other lipid traits PRSs was not shown. An epidemiological case–control study has reported a negative direct association between serum Ca concentration and knee OA through the physiological and pathological processes of chondrocytes [58]. Therefore, these previous and present study findings support the idea of a genetic overlap between decreased Ca levels and knee OA.

This study had several limitations. First, the sample size was small. Second, the R^2^ on the liability scale of each PRS was insufficiently high. This might be because the current PRSs may only partially capture the heritability of knee OA. Third, multiple array datasets were merged and analyzed; therefore, there is a risk of bias due to an imbalance in sample size and knee OA prevalence. However, when we evaluated the OR of knee OA of the MTAG-multi-population knee OA PRS for each array dataset and WGS data, we found a consistent contribution to knee OA (Supplementary Fig. S3).

In conclusion, this study showed that multi-trait PRS based on MTAG using multi-population GWAS with a large sample size was significantly associated with knee OA in the Japanese population, even when the sample size of GWAS of the same ancestry was small. To our knowledge, this is the first study to show a statistically significant association between PRS and knee OA in a non-European population.

## Supplementary Information

**Additional file 1: Supplementary Fig. 1.** Flowchart of the genotype quality control and sample extraction for PRS calculation. **Supplementary Fig. 2.** Significant genetic correlation coefficient calculated using LDSC between each risk factor trait. **Supplementary Fig. 3.** Forest plot of adjusted odds ratios of MTAG-based multi-population knee OA PRS for each genotyping array and whole genome sequence data. **Supplementary Table 1.** Correlation between PRSs and knee OA in single-PRS analysis. **Supplementary Table 2.** Summary of the PRS analysis of lipid traits in the sensitivity analysis excluding statin users. **Supplementary Table 3.** Summary of the PRS analysis of traits that showed association with knee OA using LDpred2-auto.

## Data Availability

The datasets used and/or analyzed during the current study are available from the corresponding author upon reasonable request.

## Abbreviations

AUC: Area under the curve
BBJ: Biobank Japan
BMI: Body mass index
Ca: Calcium
CI: Confidence interval
GO: Genetics of osteoarthritis
GWAS: Genome-wide association study
MAC: Minor allele count
MAF: Minor allele frequency
HDL-C: High-density lipoprotein cholesterol
QC: Quality control
HWE: Hardy-Weinberg equilibrium
K–L: Kellgren–Lawrence
LD: Linkage disequilibrium
MTAG: Multi-trait analysis of genome-wide association
NRI: Net reclassification improvement
OA: Osteoarthritis
OR: Odds ratio
PRS: Polygenic risk score
ROC: Receiver operating characteristic
SD: Standard deviation
SNP: Single-nucleotide polymorphism
TKR: Total knee replacement
TJR: Total knee and/or hip joint replacement
WGS: Whole genome sequencing
