# Supplementary material for: Improved genetic prediction of the risk of knee osteoarthritis using the risk factor-based polygenic score
Source: Arthritis Res Ther. 2023 Jun 12;25:103. doi: 10.1186/s13075-023-03082-y (PMC10258963; doi:10.1186/s13075-023-03082-y)
Supplement: Supplementary file 1 — Additional file 1: Supplementary Fig. 1. Flowchart of the genotype quality control and sample extraction for PRS calculation. Supplementary Fig. 2. Significant genetic correlation coefficient calculated using LDSC between each risk factor trait. Supplementary Fig. 3. Forest plot of adjusted odds ratios of MTAG-based multi-population knee OA PRS for each genotyping array and whole genome sequence data. Supplementary Table 1. Correlation between PRSs and knee OA in single-PRS analysis. Supplementary Table 2. Summary of the PRS analysis of lipid traits in the sensitivity analysis excluding statin users. Supplementary Table 3. Summary of the PRS analysis of traits that showed association with knee OA using LDpred2-auto. [file 13075_2023_3082_MOESM1_ESM.docx]

**Supplementary Fig. 1. Flowchart of the genotype quality control and sample extraction for PRS calculation.
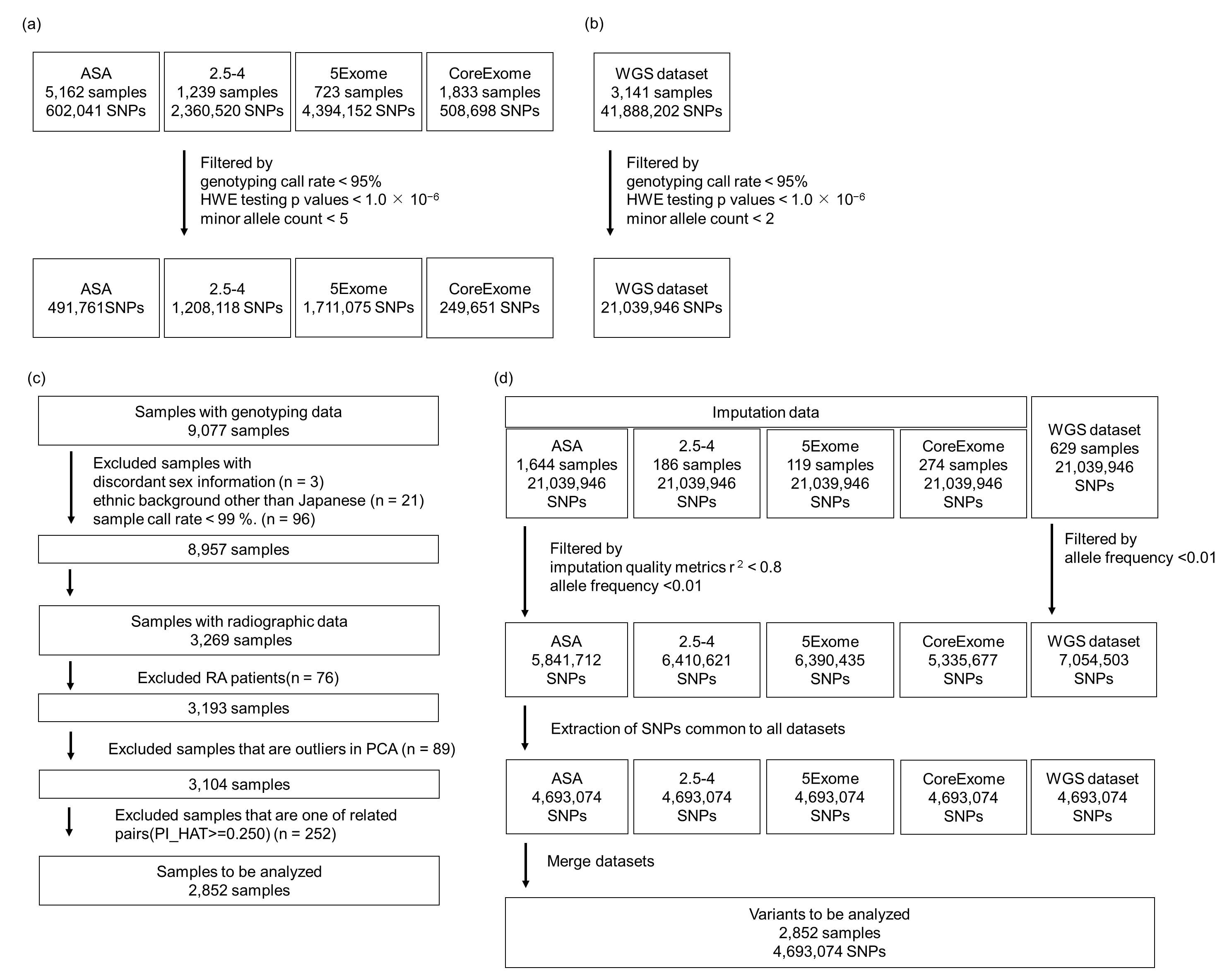
**

**Supplementary Fig. 2. Significant genetic correlation coefficient calculated using LDSC between each risk factor trait**.


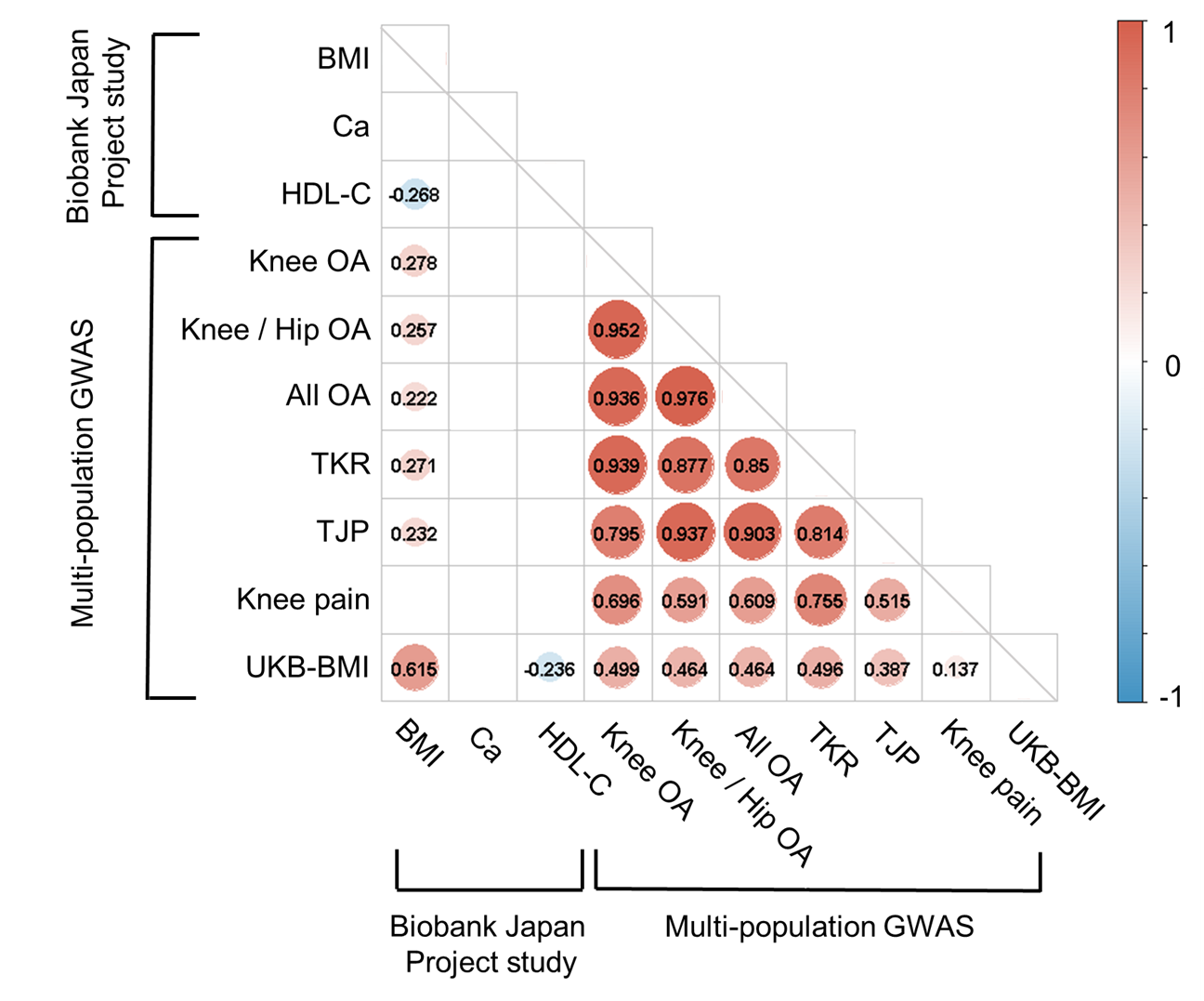


**Supplementary Fig. 3. Forest plot of adjusted odds ratios of MTAG-based multi-population knee OA PRS for each genotyping array and whole genome sequence data.**

**
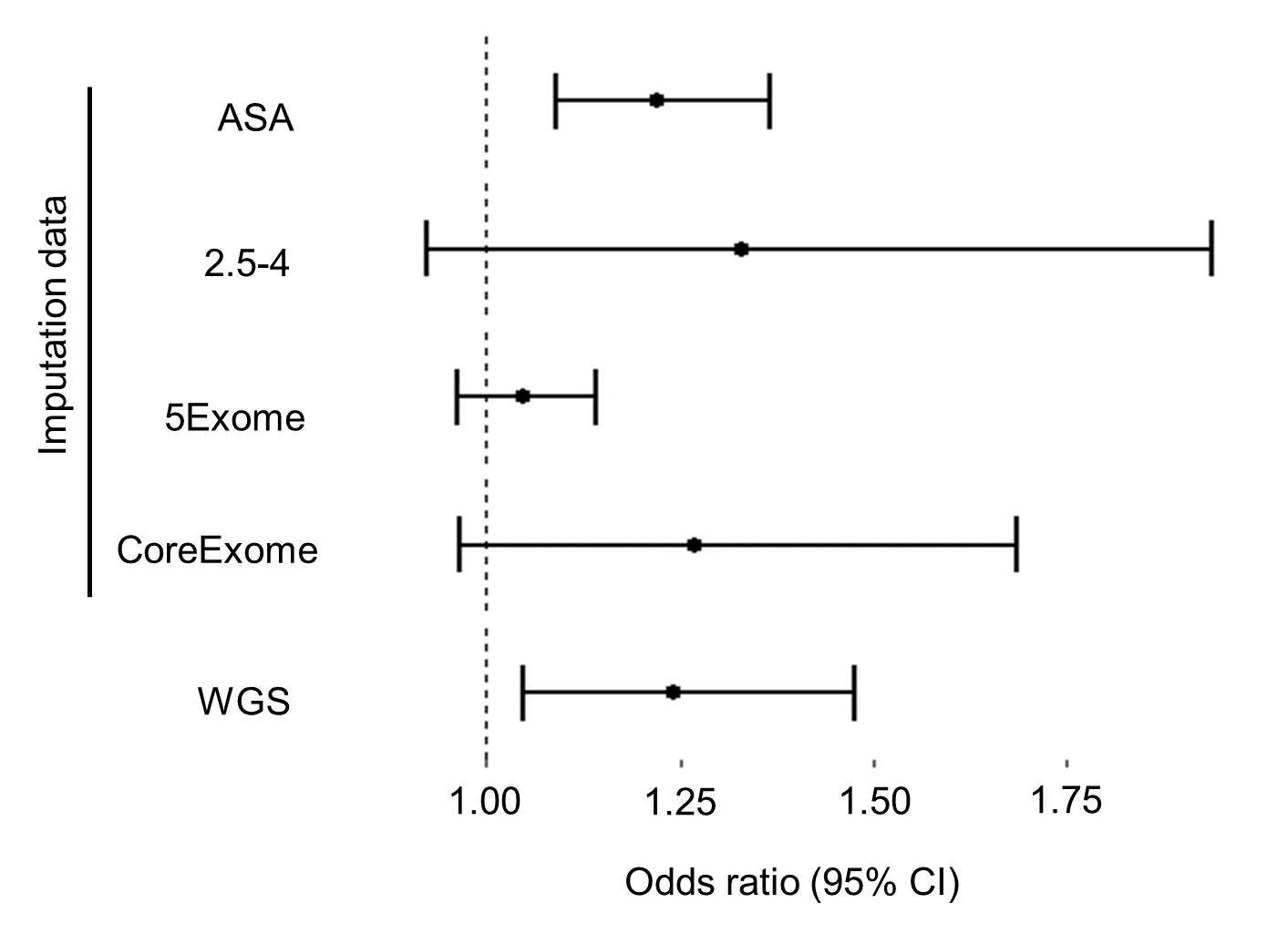
**

**Supplementary Table 1. Correlation between PRSs and knee OA in single-PRS analysis**

| Traits | | *p*-value | | R^2^ (%) | | AUC |
| --- | --- | --- | --- | --- | --- | --- |
| **OA PRS analysis** |  |  |  |  |  |  |
| **Japanese GWAS** | |  |  | |  | |
| Knee OA | | 0.228 | | 0.018 | | 0.510 |
| **Multi-population GWAS** | |  |  | |  | |
| Knee OA | | 6.70E-05 | | 0.520 | | 0.540 |
| Hip OA | | 0.296 | | 0.056 | | 0.515 |
| Knee and/or hip OA | | 9.90E-04 | | 0.421 | | 0.534 |
| Spine OA | | 0.420 | | 0.049 | | 0.515 |
| Hand OA | | 0.937 | | 0.002 | | 0.503 |
| Thumb OA | | 0.500 | | 0.060 | | 0.515 |
| All OA | | 0.014 | | 0.330 | | 0.530 |
| Total knee replacement | | 0.008 | | 0.165 | | 0.523 |
| Total hip replacement | | 0.369 | | 0.022 | | 0.492 |
| Total knee and/or hip replacement | | 0.025 | | 0.144 | | 0.524 |
| Knee pain | | 0.026 | | 0.211 | | 0.527 |
| **Risk trait PRS analysis** | |  | |  | |  |
| **Biobank Japan GWAS** | |  | |  | |  |
| Body mass index | | 4.57E-06 | | 0.689 | | 0.548 |
| Albumin/globulin ratio | | 0.966 | | 0.004 | | 0.505 |
| Alkaline phosphatase | | 0.745 | | 0.049 | | 0.515 |
| Alanine aminotransferase | | 0.053 | | 0.113 | | 0.521 |
| Activated partial thromboplastin time | | 0.927 | | <0.001 | | 0.503 |
| Aspartate aminotransferase | | 0.146 | | 0.076 | | 0.519 |
| Albumin | | 0.429 | | 0.014 | | 0.508 |
| Blood sugar | | 0.992 | | 0.009 | | 0.504 |
| Blood urea nitrogen | | 0.292 | | 0.063 | | 0.516 |
| Basophil count | | 0.555 | | 0.005 | | 0.500 |
| Creatine kinase | | 0.804 | | 0.014 | | 0.508 |
| C-reactive protein | | 0.671 | | 0.014 | | 0.507 |
| Calcium | | 0.021 | | 0.072 | | 0.514 |
| Chloride | | 0.114 | | 0.079 | | 0.519 |
| Diastolic blood pressure | | 0.322 | | 0.012 | | 0.507 |
| E/A ratio | | 0.730 | | <0.001 | | 0.503 |
| Ejection fraction | | 0.694 | | 0.040 | | 0.514 |
| Eosinophil count | | 0.146 | | 0.107 | | 0.519 |
| Fractional shortening | | 0.247 | | 0.103 | | 0.519 |
| Fibrinogen | | 0.681 | | 0.020 | | 0.508 |
| γ-glutamyl transferase | | 0.351 | | 0.009 | | 0.506 |
| High-density-lipoprotein cholesterol | | 6.94E-04 | | 0.434 | | 0.539 |
| Hemoglobin | | 0.726 | | <0.001 | | 0.503 |
| Hemoglobin A1c | | 0.367 | | 0.052 | | 0.512 |
| Hematocrit | | 0.539 | | 0.010 | | 0.504 |
| Interventricular septum thickness | | 0.091 | | 0.085 | | 0.518 |
| Potassium | | 0.997 | | 0.003 | | 0.499 |
| Lactate dehydrogenase | | 0.152 | | 0.095 | | 0.523 |
| Low-density-lipoprotein cholesterol | | 0.152 | | 0.095 | | 0.523 |
| Left ventricular internal dimension in diastole | | 0.315 | | 0.015 | | 0.506 |
| Left ventricular internal dimension | | 0.845 | | <0.001 | | 0.499 |
| Left ventricular mass | | 0.261 | | 0.046 | | 0.511 |
| Left ventricular mass index | | 0.082 | | 0.113 | | 0.518 |
| Lymphocyte count | | 0.375 | | 0.019 | | 0.509 |
| Mean arterial pressure | | 0.653 | | 0.003 | | 0.500 |
| Mean corpuscular hemoglobin | | 0.760 | | 0.038 | | 0.513 |
| Mean corpuscular hemoglobin concentration | | 0.954 | | 0.007 | | 0.507 |
| Mean corpuscular volume | | 0.812 | | 0.022 | | 0.507 |
| Monocyte count | | 0.056 | | 0.077 | | 0.513 |
| Non-albumin protein | | 0.857 | | <0.001 | | 0.497 |
| Sodium | | 0.204 | | 0.097 | | 0.517 |
| Neutrophil count | | 0.397 | | 0.091 | | 0.516 |
| Phosphorus | | 0.440 | | 0.007 | | 0.506 |
| Pulse pressure | | 0.966 | | 0.020 | | 0.503 |
| Prothrombin time | | 0.484 | | 0.004 | | 0.503 |
| Posterior wall thickness | | 0.803 | | 0.004 | | 0.505 |
| Platelet count | | 0.444 | | 0.008 | | 0.507 |
| Red blood cell count | | 0.838 | | 0.013 | | 0.504 |
| Relative wall thickness | | 0.933 | | 0.008 | | 0.495 |
| Systolic blood pressure | | 0.553 | | 0.032 | | 0.507 |
| Total bilirubin | | 0.270 | | 0.065 | | 0.489 |
| Total cholesterol | | 0.094 | | 0.108 | | 0.516 |
| Triglyceride | | 0.446 | | 0.015 | | 0.493 |
| Total protein | | 0.716 | | 0.001 | | 0.499 |
| Uric acid | | 0.791 | | 0.010 | | 0.496 |
| White blood cell count | | 0.413 | | 0.055 | | 0.511 |
| Zinc sulfate turbidity test | | 0.840 | | 0.009 | | 0.507 |
| Estimated glomerular filtration rate | | 0.223 | | 0.022 | | 0.510 |
| Serum creatinine | | 0.126 | | 0.041 | | 0.512 |
| Age of smoking initiation | | 0.100 | | 0.069 | | 0.511 |
| Cigarettes per day | | 0.423 | | 0.020 | | 0.510 |
| Height | | 0.809 | | 0.010 | | 0.494 |
| **Multi-population GWAS** | |  | |  | |  |
| UKB body mass index | | 0.002 | | 0.523 | | 0.540 |
| UKB calcium | | 0.329 | | 0.091 | | 0.524 |
| UKB high-density-lipoprotein cholesterol | | 0.282 | | 0.055 | | 0.514 |

PRS, polygenic risk score; OA, osteoarthritis; AUC, area under the curve; GWAS, genome-wide association study; UKB, UK Biobank.

*P*-values were evaluated using a logistic regression model. Age, sex, 10 principal components, and type of dataset were included as covariates. Disease liability explained by the PRS was estimated by the conversion of observed PVE to R^2^ on the liability scale using a linear model.

**Supplementary Table 2. Summary of the PRS analysis of lipid traits in the sensitivity analysis excluding statin users**

| Traits | *p*-value | Adjusted OR  (95% CI) | R^2^ (%) | AUC |
| --- | --- | --- | --- | --- |
| **Biobank Japan GWAS** |  |  |  |  |
| High-density-lipoprotein cholesterol | 0.004* | 0.86 (0.78–0.95) | 0.427 | 0.540 |
| Low-density-lipoprotein cholesterol | 0.050 | 0.90 (0.81–1.00) | 0.321 | 0.535 |
| Total cholesterol | 0.081 | 1.09 (0.99–1.21) | 0.123 | 0.519 |
| Triglyceride | 0.469 | 1.03 (0.94–1.15) | 0.004 | 0.495 |

*Significant after Bonferroni correction

PRS, polygenic risk score; GWAS, genome-wide association study.

*P*-values were evaluated using a logistic regression model. Age, sex, 10 principal components, and type of dataset were included as covariates. Disease liability explained by the PRS was estimated by the conversion of the observed PVE to R^2^ on the liability scale using a linear model.

**Supplementary Table 3. Summary of the PRS analysis of traits that showed association with knee OA using LDpred2-auto.**

| Traits | *p*-value | Adjusted OR  (95% CI) | R^2^ (%) | AUC | AUC test *p*-value | NRI (*p*-value) |
| --- | --- | --- | --- | --- | --- | --- |
| **OA PRS analysis** |  |  |  |  |  |  |
| Japanese knee OA | 0.563 | 1.02 (0.94–1.11) | 0.017 | 0.508 | 0.955 | 0.015 (0.704) |
| **Multi-population GWAS** | | |  |  |  |  |
| Knee OA | 0.004*† | 1.13 (1.04–1.23) | 0.284 | 0.527 | 0.119 | 0.046 (0.235) |
| Knee and/or hip OA | 0.005 | 1.13 (1.04–1.22) | 0.288 | 0.528 | 0.137 | 0.041 (0.294) |
| All OA | 0.030 | 1.10 (1.01–1.19) | 0.216 | 0.522 | 0.156 | 0.024 (0.539) |
| Total knee replacement | 0.054 | 1.08 (1.00–1.18) | 0.070 | 0.516 | 0.326 | 0.056 (0.151) |
| Total knee and/or hip replacement | 0.047 | 1.09 (1.00–1.18) | 0.129 | 0.521 | 0.200 | 0.042 (0.284) |
| Knee pain | 0.048 | 1.09 (1.00-1.18) | 0.197 | 0.527 | 0.177 | 0.112 (0.004) |
| **Risk trait PRS analysis** |  |  |  |  |  |  |
| **Biobank Japan GWAS** |  |  |  |  |  |  |
| Body mass index | 4.80E-05*† | 1.19 (1.09–1.29) | 0.523 | 0.544 | 0.296 | 0.085 (0.029) |
| Calcium | 0.289 | 0.96 (0.88–1.04) | 0.006 | 0.495 | 0.444 | 0.013 (0.747) |
| High-density-lipoprotein cholesterol | 0.030 | 0.91 (0.84–0.99) | 0.218 | 0.527 | 0.382 | 0.056 (0.146) |
| **Multi-population GWAS** |  |  |  |  |  |  |
| UKB-body mass index | 0.013 | 1.11 (1.02–1.21) | 0.241 | 0.530 | 0.519 | –0.006 (0.878) |

*Significant after Bonferroni correction; †Significant after Bonferroni correction in both the PRS analysis using PRScs and LDpred2-auto

PRS, polygenic risk score; OA, osteoarthritis; OR, odds ratio; AUC, area under the curve; NRI, net reclassification improvement; GWAS, genome-wide association study; UKB, UK Biobank.

*P*-values were evaluated using a logistic regression model. Adjusted OR per standard deviation and 95% confidence intervals of the risk of knee OA were calculated using a logistic regression model, including age, sex, 10 principal components, and a dummy variable for the array type used in genotyping as covariates. Disease liability explained by the PRS was estimated by the conversion of observed PVE to R^2^ on the liability scale using a linear model. The AUC test (Delong method) and net reclassification improvement analysis were performed to compare MODEL I (only clinical information, including sex, age, and BMI) and MODEL II (incorporating PRS into MODEL I).
